# Synthesis and biological evaluation of glucagon-like peptide-1 receptor agonists

**DOI:** 10.1007/s12272-013-0253-9

**Published:** 2013-11-01

**Authors:** Yu-Juan Zhang, Liu-Lan Shen, Hyae-Gyeong Cheon, Yong-Nan Xu, Jin-Hyun Jeong

**Affiliations:** 1 School of Pharmaceutical Engineering, Shenyang Pharmaceutical University, Shenyang, 110016 Liaoning China; 2 Yonsei Institute of Pharmaceutical Sciences and College of Pharmacy, Yonsei University, 162-Songdo-dong, Yeonsu-gu, Incheon, 406-840 Republic of Korea; 3 Department of Pharmacology and Pharmaceutical Sciences, Gachon University of Medicine and Science, Incheon, 406-799 Republic of Korea

**Keywords:** Small molecule agonists, GLP-1R, Heterocycles, Structure–activity relationships, Synthesis

## Abstract

In this study, a series of fused-heterocyclic derivatives were systematically designed and synthesized using an efficient route, and evaluated in terms of GLP-1R agonist activity. We employed short synthetic steps and reactions that are tolerant of the presence of various functional groups and suitable for parallel operations to enable the rapid generation of libraries of diverse and structurally complex small molecules. Of the compounds synthesized, 3-(8-chloro-6-(trifluoromethyl)imidazo[1,2-*a*] pyridin-2-yl)phenyl methanesulfonate (**8e**) was the most potent agonist with an EC_50_ of 7.89 μM, and thus is the compound with the greatest potential for application. These findings represent a valuable starting point for the design and discovery of small-molecule GLP-1R agonists that can be administered orally.

**Electronic supplementary material:**

The online version of this article (doi:10.1007/s12272-013-0253-9) contains supplementary material, which is available to authorized users.

## Introduction

Type 2 diabetes mellitus (DM2), a state of hormonal disruption and incretin deficiency, is increasingly becoming a worldwide epidemic (Kwak and Ha 2013). Current drugs utilized in the treatment of DM2 have well-established shortcomings: (1) increasing body weight and (2) increasing loss of β-cell function (Whitehouse 1997; Giugliano et al. 2009). However, the recent emergence of incretin-based therapies, which focus on glucagon-like peptide-1 (GLP-1), has attracted much interest.

GLP-1 is a peptide hormone of 30 amino acid residues. As a peptide, it has a very short half-life (<2 min) (Deacon et al. 1995). Such a short half-life has limited the utility of native GLP-1 in the treatment of DM2. The effort to identify GLP-1 analogues has resulted in the development of the drugs exenatide (Sennik et al. 2011; Buse et al. 2004) and liraglutide (Sjöholm 2010; Hribal and Sesti 2010). However, the requirement for injection limits the clinical utility of these peptide drugs. Therefore, orally active, small-molecule agonists of the GLP-1 receptor (GLP-1R) are highly sought after (Murphy and Bloom 2007).

**Fig. 1:**
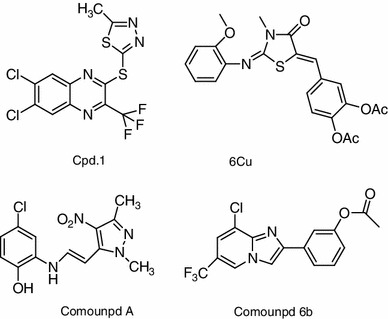
Known ago-allosteric modulators of GLP-1R

**Fig. 2:**
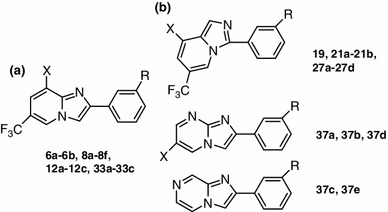
Structures of synthesized compounds. **a** Synthesized imidazo[1,2-a]pyridine-based molecules. **b** Other synthesized heterocycle-series compounds

Figure 1 shows synthetic small molecule agonists reported by several groups (Teng et al. 2000; Wang et al. 2009; Teng et al. 2007; Kopin 2004; Gong et al. 2010). Compound **6b**, characterized by a novel imidazopyridine hit core, was identified from a library of 10,000 heterocyclic small molecules (Gong et al. 2010). As a small and drug-like active molecule, it represents an interesting starting point for the development of novel drugs. Therefore, we selected this compound as a model. In an effort to move away from the labile ester group of the phenol, we planned a synthetic pathway of new derivatives of imidazo[1,2-α]pyridine-based molecules (Fig. 2). To evaluate the structure–activity relationship, we designed and synthesized a series of heterocyclic derivatives containing a ring-junction nitrogen using a three-dimensional (3D) pharmacophore model reported previously (Gong et al. 2010) (Fig. 2). For the first stage, only combinations of five- and six-membered rings are considered, including imidazo[1,5-α]pyridine, imidazo[1,2-α]pyrimidine and imidazo[1,2-α]pyrazine. We employed short synthetic steps and reactions that are tolerant of the presence of various functional groups and suitable for parallel operations to enable the rapid generation of libraries of diverse, structurally complex, small molecules.

## Materials and methods

### Chemistry

All the chemicals used in synthesis were supplied by Aldrichand TCI, and were used without further purification. All solvents were purified and stored in a dry condition. Reaction progress was determined by thin-layer chromatography (TLC) on Merck TLC Silica gel 60 F245 plates. Column chromatography was carried out using a silica gel 60 (63–200 mesh, Merck). NMR spectra were recorded on Agilent 400 instruments operating at 400 MHz for ^1^H and 100 MHz for ^13^C, and Agilent 500 instruments operating at 500 MHz for ^1^H and 125 MHz for ^13^C. Chemical shifts are expressed as parts per million (ppm) with tetramethylsilane as the internal standard. MS spectra were recorded on an Agilent G6530A Q-TOF.

### General synthetic procedure for (**6a**–**b**)

To a stirred solution of bromomethylketone **3** (1.21 g, 4.7 mmol) and 2-amino-5-trifuoromethylpyridine **4** (0.61 g, 4.7 mmol) or 2-amino-3-chloro-5-trifluoromethylpyridine **5** (0.92 g, 4.7 mmol) in EtOH (50 mL) was added NaHCO_3_ (0.31 g, 4.7 mmol) at room temperature. The reaction mixture was heated to reflux and monitored by TLC (hexane/ethyl acetate: 2/1) until completion. After removing EtOH, the residue was extracted with ethyl acetate and water. The combined organic phases were washed with water, 1 N HCl, and brine, dried, and filtered and concentrated in vacuo. The residue was purified by silica gel column chromatography (ethyl acetate/hexane = 10–20 %, Rf = 0.23).

#### 3-(6-(Trifluoromethyl)imidazo[1,2-*a*]pyridin-2-yl)phenyl acetate (**6a**)

Pale yellow solid; Yield: 64 %;^1^H NMR (500 MHz, CDCl_3_): δ 2.33 (s, 3H), 7.10 (d, *J* = 10.1 Hz, 1H), 7.32 (d, *J* = 11.9 Hz, 1H), 7.45 (t, *J* = 10.0 Hz, 1H), 7.70–7.73 (m, 2H), 7.80 (d, *J* = 9.7 Hz, 1H), 7.94(s, 1H), 8.49 (s, 1H);^13^C NMR (125 MHz, CDCl_3_); δ 21.1, 109.8, 117.0, 117.3, 118.0, 119.5, 121.1, 121.8, 123.6, 124.8, 129.9, 134.2, 145.2, 146.2, 151.2, 169.6; EI-HRMS calculated for (C_16_H_11_F_3_N_2_O_2_+H)^+^ 321.0851, found 321.0860.

#### 3-(8-Chloro-6-(trifluoromethyl)imidazo[1,2-*a*]pyridin-2-yl)phenyl acetate (**6b**)

Pale yellow solid; Yield: 29 %;^1^H NMR (500 MHz, CDCl_3_): δ 2.34 (s, 3H), 7.10 (dd, *J* = 10.1, 2.1 Hz, 1H), 7.41–7.47 (m, 2H), 7.76 (t, *J* = 2.2 Hz, 1H), 7.81 (d, *J* = 10.2 Hz, 1H), 7.98 (s, 1H), 8.43 (s, 1H);^13^C NMR (125 MHz, CDCl_3_): δ 21.2,111.2, 119.62, 119.65, 119.68, 122.0, 123.3, 123.4, 123.7, 124.3, 129.8, 134.0, 142.7, 147.0, 151.1, 169.6; EI-HRMS calculated for (C_16_H_10_ClF_3_N_2_O_2_+H)^+^ 355.0461, found 355.0470.

### General synthetic procedure for (**8a** and **8d**)

To a mixture of **7a** (99 mg, 0.36 mmol) or **7b** (113 mg, 0.36 mmol) and K_2_CO_3_ (250 mg, 1.81 mmol) in acetone (10 mL) was added 1-chloroacetone (1 mL, 34.83 mmol) at room temperature. The reaction mixture was heated to reflux for 6 h. After removing acetone and 1-chloroacetone, the residue was extracted with ethyl acetate and water. The combined organic phases were washed with water and brine, dried, and filtered and concentrated in vacuo. The residue was purified by silica gel column chromatography (ethyl acetate/hexane = 10–20 %, Rf = 0.25).

### General synthetic procedure for (**8b** and **8e**)

To a solution of **7a** (98 mg, 0.35 mmol) or **7b** (121 mg, 0.45 mmol) in pyridine (5 mL) was added methanesulfonyl chloride (66 mg, 0.60 mmol) dropwise with stirring overnight in an ice bath. The reaction mixture was quenched with water in an ice bath and extracted with ethyl acetate (3 × 30 mL). The combined organic phases were washed with water, 1 N HCl, and brine, dried, and filtered and concentrated in vacuo. The residue was purified by silica gel column chromatography (hexane/ethyl acetate = 4/1, Rf = 0.25).

### General synthetic procedure for (**8c** and **8f**)

To a solution of **7a** (90 mg, 0.32 mmol) or **7b** (100 mg, 0.32 mmol) in pyridine (10 mL) was added toluenesulfonyl chloride (80 mg, 0.42 mmol) dropwise in an ice bath. After stirring for 2 h at room temperature, the reaction mixture was quenched with water in an ice bath and then extracted with ethyl acetate (3 × 30 mL). The combined organic phases were washed with water, 1 N HCl, and brine, dried, and filtered and concentrated. The residue was purified by silica gel column chromatography (hexane/ethyl acetate = 8/1, Rf = 0.23).

#### 1-(3-(6-(Trifluoromethyl)imidazo[1,2-*a*]pyridin-2-yl)phenoxy)propan-2-one (**8a**)

Pale yellow solid; Yield: 63 %;^1^H NMR (500 MHz, CDCl_3_): δ 2.32 (s, 3H), 4.64 (s, 2H), 6.91 (d, *J* = 10.1 Hz, 1H), 7.32–7.40 (m, 2H), 7.55–7.57 (m, 2H), 7.73 (d, *J* = 11.8 Hz, 1H), 7.94 (s, 1H), 8.50 (s, 1H);^13^C NMR (125 MHz, CDCl_3_): δ 26.7, 73.0, 109.6, 112.2, 114.8, 118.1, 119.6, 120.7, 122.4, 124.6, 126.7, 130.1, 134.6, 145.2, 147.1, 158.2, 205.5; EI-HRMS calculated for (C_17_H_13_F_3_N_2_O_2_+H)^+^ 335.1007, found 335.1019.

#### 3-(6-(Trifluoromethyl)imidazo[1,2-*a*]pyridin-2-yl)phenyl methanesulfonate (**8b**)

Pale yellow solid; Yield: 44 %;^1^H NMR (500 MHz, CDCl_3_): δ 3.20 (s, 3H), 7.29–7.36 (m, 2H), 7.50 (t, *J* = 9.9 Hz, 1H), 7.72 (d, *J* = 11.7 Hz, 1H), 7.89–7.92 (m, 2H), 7.98 (s, 1H), 8.51 (s, 1H);^13^C NMR (125 MHz, CDCl_3_): δ 37.6, 109.9, 118.3, 119.7, 121.09, 121.11, 122.0, 124.77, 124.81, 125.0, 130.5, 135.3, 145.3, 145.9, 149.8; EI-HRMS calculated for (C_15_H_11_F_3_N_2_O_3_S + Na)^+^ 379.0340, found 379.0360.

#### 3-(6-(Trifluoromethyl)imidazo[1,2-*a*]pyridin-2-yl)phenyl 4-methylbenzenesulfonate (**8c**)

Pale yellow solid; Yield: 64 %;^1^H NMR (500 MHz, CDCl_3_): δ 2.43 (s, 3H), 6.91 (d, *J* = 8.2 Hz, 1H), 7.30–7.35 (m, 4H), 7.62 (s, 1H), 7.67 (m, 1H), 7.75 (d, *J* = 7.4 Hz, 2H), 7.84–7.89 (m, 2H), 8.48(s, 1H);^13^C NMR (125 MHz, CDCl_3_): δ 21.7, 109.8, 118.2, 120.2, 120.9, 122.0, 124.8, 128.5, 129.8, 130.0, 132.2, 134.9, 145.3, 145.5, 146.1, 150.1; EI-HRMS calculated for (C_21_H_15_F_3_N_2_O_3_S + Na)^+^ 455.0653, found 455.0656.

#### 1-(3-(8-Chloro-6-(trifluoromethyl)imidazo[1,2-*a*]pyridin-2-yl)phenoxy)propan-2-one (**8d**)

Pale yellow solid; Yield: 50 %;^1^H NMR (500 MHz, CDCl_3_): δ 2.32 (s, 3H), 4.64 (s, 2H), 6.89 (dd, *J* = 8.2, 2.7 Hz, 1H), 7.35 (t, *J* = 7.9 Hz, 1H), 7.40 (s, 1H), 7.54–7.57 (m, 2H), 7.97 (s, 1H), 8.43 (s, 1H);^13^C NMR (125 MHz, CDCl_3_): δ 26.8, 73.0, 111.3, 112.6, 115.0, 116.6, 116.9, 119.6, 119.7, 123.3, 124.3, 130.1, 134.1, 142.7, 147.6, 158.1, 205.5; EI-HRMS calculated for (C_17_H_12_ClF_3_N_2_O_2_+H)^+^ 369.0618, found 369.0669.

#### 3-(8-Chloro-6-(trifluoromethyl)imidazo[1,2-*a*]pyridin-2-yl)phenyl methanesulfonate (**8e**)

Pale yellow solid;Yield: 45 %;^1^H NMR (500 MHz, CDCl_3_): δ 3.20 (s, 3H), 7.31 (d, *J* = 10.2 Hz, 1H), 7.43 (s, 1H), 7.50 (t, *J* = 10.0 Hz, 1H), 7.91 (m, 1H), 7.94 (d, *J* = 9.8 Hz, 1H), 8.03 (s, 1H), 8.45(s, 1H);^13^C NMR (125 MHz, CDCl_3_): δ 37.7, 111.5, 116.9, 117.2, 120.0, 122.2, 123.38, 123.43, 124.5, 125.3, 130.4, 134.8, 142.8, 146.4, 149.7; EI-HRMS calculated for (C_15_H_10_ClF_3_N_2_O_3_S+H)^+^ 391.0131, found 391.0135.

#### 3-(8-Chloro-6-(trifluoromethyl)imidazo[1,2-*a*]pyridin-2-yl)phenyl 4-methylbenzene sulfonate (**8f**)

Pale yellow solid; Yield: 38 %;^1^H NMR (500 MHz, CDCl_3_): δ 2.43 (s, 3H), 6.91 (d, *J* = 8.2 Hz, 1H), 7.27–7.33 (m, 3H), 7.39 (m, 1H), 7.61 (s, 1H), 7.74 (d, *J* = 8.2 Hz, 2H), 7.87 (d, *J* = 7.8 Hz, 1H), 7.94 (s, 1H), 8.48(s, 1H);^13^C NMR (125 MHz, CDCl_3_): δ 21.7, 111.5, 119.8, 120.3, 122.2, 123.5, 124.3, 125.1, 126.9, 128.5, 130.0, 132.3, 134.4, 138.6, 145.6, 145.8, 146.5, 149.8, 150.0. EI-HRMS calculated for (C_21_H_14_ClF_3_N_2_O_3_S + Na)^+^ 489.0263, found 489.0274.

### General synthetic procedure for (**12a**–**c**)

Pyridium bromide perbromide (1.79 g, 5.60 mmol) was added to a solution of **10a**–**c** (0.9 g, 5.08 mmol) in AcOH (100 mL) with stirring for 3 h at room temperature. The reaction mixture was poured into ice-cold water and then extracted with ethyl acetate (3 × 50 mL). The combined organic phases were washed with saturated aqueous NaHCO_3_, water, and brine, dried, and filtered and concentrated in vacuo to give crude **11a**–**c** as a yellow oil (1.29 g, 98 %). The resulting crude **11a**–**c** could be used without further purification. To a stirred solution of bromomethylketone **11a**–**c** (1.30 g, 5.1 mmol) and aminopyridine **4** (0.82 g, 5.1 mmol) in EtOH (80 mL) was added NaHCO_3_ (0.43 g, 5.1 mmol) at room temperature. The reaction mixture was heated to reflux for 8 h. After removing EtOH, the residue was extracted with ethyl acetate and water. The combined organic phases were washed with water, 1 N HCl, and brine, dried, and filtered and concentrated in vacuo. The residue was purified by silica gel column chromatography **(12a**–**b**, hexane/ethyl acetate = 1/1, Rf = 0.24; **12c**, hexane/ethyl acetate = 4/1, Rf = 0.22).

#### *N*-(3-(6-(Trifluoromethyl)imidazo[1,2-*a*]pyridin-2-yl)phenyl)acetamide(**12a**)

Pale yellow solid;Yield: 39 %;^1^H NMR (500 MHz, CDCl_3_): δ 2.19 (s, 3H), 7.31 (d, *J* = 11.6 Hz, 1H), 7.39 (t, *J* = 9.8 Hz, 1H), 7.51 (s, NH), 7.60 (d, *J* = 9.8 Hz, 1H), 7.68 (d, *J* = 11.1 Hz, 2H), 7.93 (s, 1H), 8.07 (s, 1H), 8.47(s, 1H);^13^C NMR (125 MHz, CDCl_3_): δ 24.7, 109.6, 117.4, 118.1, 119.9, 120.7, 122.0, 124.6, 124.7, 129.6, 133.7, 138.5, 145.2, 147.1, 168.5; EI-HRMS calculated for (C_16_H_12_F_3_N_3_O + Na)^+^ 342.0830, found 342.0835.

#### *N*-(3-(6-(Trifluoromethyl)imidazo[1,2-*a*]pyridin-2-yl)phenyl)methanesulfonamide (**12b**)

Pale yellow solid;Yield: 20 %;^1^H NMR (500 MHz, CDCl_3_): δ 3.05 (s, 3H), 7.30 (dt, *J* = 7.0, 1.3 Hz, 1H), 7.34 (dd, *J* = 9.5, 1.8 Hz, 1H), 7.40 (t, *J* = 7.9 Hz, 1H), 7.69–7.74 (m, 3H), 7.82 (t, *J* = 1.9 Hz, 1H), 7.99 (s, 1H), 8.51(s, 1H);^13^C NMR (125 MHz, CDCl_3_): δ 39.4, 110.0, 118.0, 118.5, 120.7, 121.3, 123.0, 124.8, 124.9, 130.2, 130.3, 134.2, 137.6, 145.2, 146.2; EI-HRMS calculated for (C_15_H_12_F_3_N_3_O_2_S+H)^+^ 356.0681, found 356.0705.

#### 4-Methyl-*N*-(3-(6-(trifluoromethyl)imidazo[1,2-*a*]pyridin-2-yl)phenyl)benzene sulfonamide (**12c**)

Pale yellow solid;Yield: 21 %;^1^H NMR (500 MHz, CDCl_3_): δ 2.30 (s, 3H), 7.14–7.19 (m, 3H), 7.26–7.29 (m, 2H), 7.63 (dd, *J* = 7.7, 0.9 Hz, 1H), 7.66–7.70 (m, 4H), 7.85 (brs, NH), 7.89 (s, 1H), 8.45(s, 1H);^13^C NMR (125 MHz, CDCl_3_): δ 21.5, 109.9, 118.0, 118.8, 120.7, 121.0, 122.7, 124.7, 127.3, 129.7, 129.9, 134.0, 136.0, 137.5, 143.9, 145.2, 146.5; EI-HRMS calculated for (C_21_H_16_ClF_3_N_3_O_2_S+H)^+^ 432.0994, found 432.1020.

#### 3-(8-Chloro-6-(trifluoromethyl)imidazo[1,5-*a*]pyridin-3-yl)phenyl acetate (**19**)

To a solution of **18** (300 mg, 0.80 mmol) in benzene (10 mL) was added POCl_3_ (1.2 mL, 13.04 mmol) dropwise at room temperature. The reaction mixture was heated to reflux for 6 h. After cooling to room temperature, the mixture was poured into iced-water and then extracted with ethyl acetate (3 × 50 mL). The combined organic phases were washed with water and brine, dried, and filtered and concentrated in vacuo. The residue was purified by silica gel column chromatography (hexane/ethyl acetate = 12/1, Rf = 0.25). White solid; Yield: 90 %;^1^H NMR (400 MHz, CDCl_3_): δ 2.35 (s, 3H), 6.92 (s, 1H), 7.25 (d, *J* = 8.6 Hz, 1H), 7.54–7.61 (m, 3H), 7.77 (s, 1H), 8.51(s, 1H); ^13^C NMR (100 MHz, CDCl_3_): δ 21.1, 113.9, 117.6, 117.9, 119.6, 121.5, 121.8, 122.5, 122.9, 124.9, 125.3, 126.5, 126.8, 129.7, 130.1, 130.5, 151.3, 169.2; EI-HRMS calculated for (C_16_H_10_ClF_3_N_2_O_2_+H)^+^ 355.0461, found 355.0474.

#### 3-(8-Chloro-6-(trifluoromethyl)imidazo[1,5-*a*]pyridin-3-yl)phenyl cyclohexane carboxylate (**21a**)

To a solution of **19** (257 mg, 0.72 mmol) in THF (20 mL) was added a solution of NaOH (50 mg, 1.25 mmol) in water (10 mL) with stirring for 3 h at room temperature. After removing THF, the resulting mixture was extracted with ethyl acetate. The combined organic phases were washed with water and brine, dried, and filtered and concentrated in vacuo. The resulting crude **20** could be used without further purification. Cyclohexanecarboxylic chloride (28 mg, 0.19 mmol) was added to a solution of **20** (50 mg, 0.16 mmol), TEA (19 mg, 0.19 mmol), and DMAP (4 mg, 0.03 mmol) in anhydrous CH_2_Cl_2_ (20 mL) slowly in an ice bath. After stirring for 3 h at room temperature, the reaction mixture was poured into ice water and then extracted with CH_2_Cl_2_ (3 × 20 mL). The combined organic phases were washed with 1 N HCl, water, and brine, dried, and filtered and concentrated in vacuo. The residue was purified by silica gel column chromatography (hexane/ethyl acetate = 20/1, Rf = 0.23) to afford **21a** as a pale yellow solid (64 mg, 94 %). ^1^H NMR (400 MHz, CDCl_3_): δ 1.25–1.39 (m, 4H), 1.57–1.65 (m, 2H), 1.81–1.84 (m, 2H), 2.06–2.09 (m, 2H), 2.58 (t, *J* = 10.1 Hz, 1H), 6.92 (s, 1H), 7.24 (m, 1H), 7.52–7.59 (m, 3H), 7.78 (s, 1H), 8.52(s, 1H);^13^C NMR (100 MHz, CDCl_3_): δ 25.3, 25.6, 28.8, 43.1, 113.9, 117.7, 119.6, 121.4; EI-HRMS calculated for (C_21_H_18_ClF_3_N_2_O_2_ + Na)^+^ 445.0907, found 445.0907.

#### 1-(3-(8-Chloro-6-(trifluoromethyl)imidazo[1,5-*a*]pyridin-3-yl)phenoxy)propan-2-one (**21b**)

Using the same method as for the preparation of **8a**, starting with **20** (73 mg, 0.23 mmol), 1-chloroacetone (0.5 mL, 17.41 mmol) and K_2_CO_3_ (161 mg, 1.17 mmol), **21b** was generated as a pale yellow solid (30 mg, 35 %). ^1^H NMR (400 MHz, CDCl_3_): δ 2.30 (s, 3H), 4.66 (s, 2H), 6.91 (s, 1H), 7.06 (d, *J* = 7.9 Hz, 1H), 7.28 (s, 1H), 7.35 (d, *J* = 7.5 Hz, 1H), 7.50 (d, *J* = 8.0 Hz, 1H), 7.76 (s, 1H), 8.48 (s, 1H);^13^C NMR (100 MHz, CDCl_3_): δ 26.6, 73.0, 113.9, 114.6, 116.5, 117.5, 117.9, 119.6, 120.9, 121.4, 122.1, 124.1, 126.5, 129.6, 130.2, 130.6, 141.3, 158.5, 204.6; EI-HRMS calculated for (C_17_H_12_ClF_3_N_3_O_2_+H)^+^ 369.0618, found 369.0670.

### General synthetic procedure for (**27a**–**d**)

POCl_3_ (0.3 mL, 3.40 mmol) was added to a mixture of **24** or **26a**–**c** (0.17 mmol) and pyridine (0.93 mL, 11.60 mmol) in anhydrous dichloroethane (14 mL) at room temperature. The reaction mixture was heated to reflux for 7 h. After cooling to room temperature, the reaction mixture was concentrated, filtered, and extracted with ethyl acetate. The combined organic phases were washed with 1 N HCl, water, and brine, dried, and filtered and concentrated in vacuo. The residue was purified by silica gel column chromatography (hexane/ethyl acetate = 2/1, Rf = 0.24).

#### *N*-(3-(8-Chloro-6-(trifluoromethyl)imidazo[1,5-*a*]pyridin-3-yl)phenyl)acetamide (**27b**)

Pale yellow solid; Yield: 96 %;^1^H NMR (400 MHz, CDCl_3_): δ 2.17 (s, 3H), 6.90 (s, 1H), 7.44–7.48 (m, 2H), 7.63 (d, *J* = 6.7 Hz, 1H), 7.74 (s, 1H), 7.96 (s, 1H), 8.16 (s, 1H), 8.53(s, 1H);^13^C NMR (100 MHz, CDCl_3_): δ 24.5, 113.9, 117.5, 117.9, 119.7, 119.8, 121.1, 121.5, 122.1, 123.6, 124.2, 126.4, 129.2, 129.6, 130.0, 139.1, 141.5, 168.9; EI-HRMS calculated for (C_16_H_11_ClF_3_N_3_O + Na)^+^ 376.0440, found 376.0447.

#### *N*-(3-(8-Chloro-6-(trifluoromethyl)imidazo[1,5-*a*]pyridin-3-yl)phenyl)cyclohexane carboxamide (**27c**)

Pale yellow solid; Yield: 32 %;^1^H NMR (400 MHz, CDCl_3_): δ 1.28–1.35 (m, 2H), 1.50–1.59 (m, 2H), 1.71 (m, 2H), 1.83–1.85 (m, 2H), 1.94–1.97 (m, 2H), 2.26 (t, *J* = 11.6 Hz, 1H), 6.90 (s, 1H), 7.44–7.52 (m, 2H), 7.55 (s, 1H), 7.71 (d, *J* = 7.6 Hz, 1H), 7.75 (s, 1H), 7.95 (s, 1H), 8.54 (s, 1H);^13^C NMR (100 MHz, CDCl_3_): δ 25.6, 29.6, 46.5, 113.8, 117.4, 117.8, 119.7, 119.8, 121.0, 122.2, 123.3, 126.3, 129.4, 129.6, 130.0, 139.1, 141.5, 174.6; EI-HRMS calculated for (C_21_H_19_ClF_3_N_3_O + Na)^+^ 444.1066, found 444.1075.

#### 1-(3-(8-Chloro-6-(trifluoromethyl)imidazo[1,5-*a*]pyridin-3-yl)phenyl)pyrrolidine-2,5-dione (**27d**)

Pale yellow solid; Yield: 28 %;^1^H NMR (400 MHz, CDCl_3_): δ 2.95 (s, 4H), 6.93 (s, 1H), 7.52 (d, *J* = 7.9 Hz, 1H), 7.69 (t, *J* = 7.8 Hz, 1H), 7.76–7.82 (m, 3H), 8.63 (s, 1H);^13^C NMR (100 MHz, CDCl_3_): δ 28.4, 114.0, 117.7, 118.0, 119.7, 121.5, 122.6, 124.2, 125.7, 126.4, 127.1, 128.2, 129.8, 130.3, 132.7, 140.7, 175.8; EI-HRMS calculated for (C_18_H_11_ClF_3_N_3_O_2_+H)^+^ 394.0570, found 394.0608.

#### *tert*-Butyl (3-(8-chloro-6-(trifluoromethyl)imidazo[1,5-*a*]pyridin-3-yl)phenyl) carbamate (**27a**)

Pale yellow solid;Yield: 13 %;^1^H NMR (400 MHz, CDCl_3_): δ 1.53 (s, 9H), 6.71 (s, 1H), 6.90 (s, 1H), 7.41–7.48 (m, 3H), 7.76 (s, 1H), 7.88 (s, 1H), 8.58 (s, 1H);^13^C NMR (100 MHz, CDCl_3_): δ 28.2, 113.7, 117.3, 117.6, 118.3, 119.7, 119.9, 122.3, 122.5, 124.2, 126.3, 129.5, 130.0, 139.4, 141.6, 152.6; EI-HRMS calculated for (C_19_H_17_ClF_3_N_3_O_2_ + Na)^+^ 434.0859, found 434.0865.

#### *N*-(3-(8-Chloro-6-(trifluoromethyl)imidazo[1,2-*a*]pyridin-2-yl)phenyl)acetamide (33a)

Acetic anhydride (20 mg, 0.20 mmol) was added to a mixture of **32** (50 mg, 0.16 mmol) and DMAP (3 mg, 0.02 mmol) in anhydrous CH_2_Cl_2_ (10 mL) with stirring for 1 h at room temperature. After removing the solvent, the residue was extracted with ethyl acetate and water. The combined organic phases were washed with 1 N HCl, saturated Na_2_CO_3_, water, and brine, dried, and filtered and concentrated in vacuo. The residue was purified by silica gel column chromatography (ethyl acetate/hexane = 33–50 %, Rf = 0.23). Pale yellow solid; Yield: 50 %;^1^H NMR (400 MHz, CDCl_3_): δ 2.19 (s, 3H), 7.35–7.39 (m, 2H), 7.62–7.66 (m, 2H), 7.72 (s, 1H), 7.94 (s, 1H), 8.06 (s, 1H), 8.39 (s, 1H);^13^C NMR (100 MHz, CDCl_3_): δ 24.6, 111.3, 116.6, 117.6, 119.6, 120.3, 121.5, 122.2, 123.4, 124.2, 129.5, 133.1, 138.5, 142.6, 147.5, 168.8; EI-HRMS calculated for (C_16_H_11_ClF_3_N_3_O + Na)^+^ 376.0440, found 376.0453.

### General synthetic procedure for (**33b**–**c**)

Cyclohexanecarboxylic chloride (28 mg, 0.19 mmol) or toluenesulfonyl chloride (39 mg, 0.21 mmol) was added to a solution of **32** (50 mg, 0.16 mmol), TEA (19 mg, 0.19 mmol), and DMAP (4 mg, 0.03 mmol) in anhydrous CH_2_Cl_2_ (10 mL) slowly in an ice bath. After stirring for 3 h at room temperature, the reaction mixture was poured into ice water and then extracted with CH_2_Cl_2_ (3 × 20 mL). The combined organic phases were washed with 1 N HCl, water, and brine, dried, and filtered and concentrated in vacuo. The residue was purified by silica gel column chromatography (hexane/ethyl acetate = 9/1, Rf = 0.22).

#### *N*-(3-(8-Chloro-6-(trifluoromethyl)imidazo[1,2-*a*]pyridin-2-yl)phenyl)cyclohexane carboxamide (**33b**)

Pale yellow solid; Yield: 54 %; ^1^H NMR (400 MHz, CDCl_3_): δ 1.28–1.33 (m, 2H), 1.51–1.60 (m, 2H), 1.72 (m, 2H), 1.84–1.86 (m, 2H), 1.96–1.99 (m, 2H), 2.25 (m, 1H), 7.35–7.39 (m, 2H), 7.48 (s, 1H), 7.66 (d, *J* = 7.0 Hz, 2H),7.98 (s, 1H), 8.13 (s, 1H), 8.40 (s, 1H);^13^C NMR (100 MHz, CDCl_3_): δ 25.6, 25.7, 29.7, 46.6, 111.3, 116.6, 116.9, 117.5, 119.6, 120.2, 122.0, 123.3, 124.2, 129.5, 133.0, 138.7, 142.7, 147.6, 174.7; EI-HRMS calculated for (C_21_H_19_ClF_3_N_3_O+H)^+^ 422.12470, found 422.12446.

#### *N*-(3-(8-Chloro-6-(trifluoromethyl)imidazo[1,2-*a*]pyridin-2-y l)phenyl)-4-toluenesulfonamide (**33c**)

Pale yellow solid;Yield: 68 %;^1^H NMR (400 MHz, CDCl_3_): δ 2.46 (s, 3H), 7.00 (d, *J* = 7.5 Hz, 1H), 7.33–7.35 (m, 3H), 7.40 (s, 1H), 7.58 (s, 1H), 7.83–7.87 (m, 4H), 8.10 (d, *J* = 7.4 Hz, 1H), 8.40 (s, 1H);^13^C NMR (100 MHz, CDCl_3_): δ 21.7, 111.3, 116.6, 117.0, 119.7, 123.3, 124.4, 128.2, 128.5, 128.6, 129.4, 129.6, 129.7, 129.8, 131.5, 133.9, 134.9, 136.5, 142.7, 145.1, 146.6; EI-HRMS calculated for (C_21_H_15_ClF_3_N_3_O_2_S+H)^+^ 466.06038, found 466.07034.

### General synthetic procedure for (**37a**–**c**)

A mixture of 5-bromopyrimidin-2-amine **35**–**36** (222 mg, 1.27 mmol) and bromoacetone **3** (257 mg, 1.0 mmol) in dioxane (10 mL) with or without NaHCO_3_ (84 mg, 1.00 mmol) was stirred until reflux for 7 h. After cooling to room temperature, ethyl acetate was added, washed with water and brine, dried over calcium oxide, and filtered and concentrated in vacuo. The residue was purified by silica gel column chromatography (gradient eluent: ethyl acetate/hexane = 20–50 %, Rf = 0.21).

#### 3-(6-Bromoimidazo[1,2-*a*]pyrimidin-2-yl)phenyl acetate (37a)

White solid;Yield: 11 %; ^1^H NMR (400 MHz, CDCl_3_): δ 2.33 (s, 3H), 7.10 (d, *J* = 7.9 Hz, 1H), 7.45 (t, *J* = 7.9 Hz, 1H), 7.75 (s, 1H), 7.77 (s, 1H), 7.84 (d, *J* = 7.6 Hz, 1H), 8.51 (s, 1H), 8.55 (s, 1H);^13^C NMR (100 MHz, CDCl_3_): δ 21.2, 104.8, 106.6, 119.6, 122.1, 123.7, 129.8, 132.5, 134.2, 150.7, 151.2, 158.6, 169.5; EI-HRMS calculated for (C_14_H_10_BrN_3_O_2_+H)^+^ 332.00346, found 332.00372.

#### 3-(Imidazo[1,2-*a*]pyrimidin-2-yl)phenyl acetate (**37b**)

Pale yellow solid; Yield: 21 %;^1^H NMR (400 MHz, CDCl_3_): δ 2.32 (s, 3H), 6.82 (t, *J* = 5.6 Hz, 1H), 7.07 (d, *J* = 7.7 Hz, 1H), 7.42 (t, *J* = 7.8 Hz, 1H), 7.76 (m, 2H), 7.83 (d, *J* = 7.7 Hz, 1H), 8.39 (d, *J* = 6.3 Hz, 1H), 8.49 (s, 1H);^13^C NMR (100 MHz, CDCl_3_): δ 21.2, 106.5, 108.9, 119.5, 121.7, 123.6, 129.7, 133.1, 134.7, 146.2, 148.6, 150.1, 151.2, 169.5; EI-HRMS calculated for (C_14_H_11 N3_O_2_+H)^+^ 254.09295, found 254.09322.

#### 3-(Imidazo[1,2-*a*]pyrazin-2-yl)phenyl acetate (**37c**)

Pale yellow solid;Yield: 11 %;^1^H NMR (400 MHz, CDCl_3_): δ 2.34 (s, 3H), 7.12 (d, *J* = 7.6 Hz, 1H), 7.47 (t, *J* = 7.5 Hz, 1H), 7.74 (s, 1H), 7.83 (d, *J* = 7.3 Hz, 1H), 7.89 (s, 1H), 7.95 (s, 1H), 8.07 (s, 1H), 9.10 (s, 1H);^13^C NMR (100 MHz, CDCl_3_): δ 21.1, 109.4, 118.6, 119.6, 122.0, 123.7, 129.8, 129.9, 134.4, 140.9, 143.8, 146.8, 151.2, 169.4; EI-HRMS calculated for (C_14_H_11 N3_O_2_+H)^+^ 254.09295, found 254.09288.

#### 2-(3-Nitrophenyl)imidazo[1,2-*a*]pyrimidine (**37d**)

A mixture of 2-aminopyrazine **34** (195 mg, 2.05 mmol) and bromoacetone **29** (660 mg, 2.70 mmol) in ethanol (20 mL) was stirred until reflux for 3 h, After cooling to room temperature, the mixture was concentrated. The residue was dissolved in ethyl acetate, washed with 1 N HCl, water, and brine, dried over calcium oxide, and filtered and concentrated in vacuo. The residue was purified by silica gel column chromatography (methanol/methylene chloride = 0–1 %, Rf = 0.21) to afford **37d** as a yellow solid (24 mg, 5 %). ^1^H NMR (400 MHz, CDCl_3_): δ 6.95 (t, *J* = 1.7 Hz, 1H), 7.65 (t, *J* = 8.0 Hz, 1H), 7.97 (s, 1H), 8.21 (d, *J* = 7.9 Hz, 1H), 8.45 (d, *J* = 7.6 Hz, 1H), 8.50 (d, *J* = 5.9 Hz, 1H), 8.61 (d, *J* = 1.2 Hz, 1H), 8.80 (s, 1H);^13^C NMR (100 MHz, CDCl_3_): δ 107.1, 108.8, 109.4, 113.6, 113.9, 120.9, 123.1, 129.9, 132.2, 133.3, 134.9, 150.8; EI-HRMS calculated for (C_12_H_8_N_4_O_2_+H)^+^ 241.07255, found 241.07288.

#### 2-(3-Nitrophenyl)imidazo[1,2-*a*]pyrazine (37e)

A mixture of 2-aminopyrazine **36** (95 mg, 1.0 mmol) and bromoacetone **29** (488 mg, 2.0 mmol) in ethanol (10 mL) was stirred until reflux for 3 h. After cooling to room temperature, the mixture was concentrated. Then the residue was dissolved in ethyl acetate and washed with water. The combined aqueous phase was extracted with ethyl acetate. The combined organic phase was washed with brine, dried over calcium oxide, and filtered and concentrated in vacuo. The residue was purified by silica gel column chromatography (methylene chloride/methanol = 100/1, Rf = 0.21) to afford **37e** as a yellow solid (40 mg, 17 %). ^1^H NMR (400 MHz, CDCl_3_): δ 7.68 (t, *J* = 7.6 Hz, 1H), 7.97 (s, 1H), 8.11 (s, 1H), 8.13 (s, 1H), 8.24 (d, *J* = 7.3 Hz, 1H), 8.37 (d, *J* = 6.7 Hz, 1H), 8.80 (s, 1H), 9.18 (s, 1H);^13^C NMR (100 MHz,CDCl_3_): δ 102.6, 110.0, 114.3, 117.1, 118.9, 121.2, 123.4, 129.7, 130.1, 132.2, 134.6, 144.1; EI-HRMS calculated for (C_12_H_8_N_4_O_2_+H)^+^ 241.07255, found 241.07260.

### Biology

#### In vitro GLP-1R activation assay (Chen et al. 2007)

CHO-K1 cells (4 × 10^6^/100 mm dish) were transiently transfected with the pCMV6-GLP-1R (Origene #SC124060) and pCRE-Luc (Promega #631911) plasmids using Lipofectamine 2000 (Invitrogen, Carlsbad, CA, USA). After 24 h incubation at 37 °C, cells were seeded into 96-well culture plates (2 × 10^4^/well), and further incubated at 37 °C overnight. At the time of assay, GLP-1 (7–37) (Sigma, St. Louis, MO) or test compounds in DMSO were added to the plate. After 8 h incubation, cells were lysed and luciferase activity quantified using the Steady-Glo luciferase assay system (Promega #E2550).

Data were analyzed in Excel and EC_50_ values were determined graphically from dose–response curves in OriginPro.

## Results and discussion

### Chemistry

**Scheme 1:**
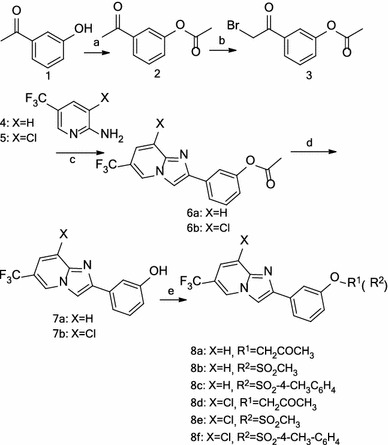
Reagents and conditions: (*a*) acetic anhydride, DMAP, anhydrous CH_2_Cl_2_, RT; (*b*) Br_2_, AlCl_3_, Et_2_O, ice bath; (*c*) NaHCO_3_, EtOH, reflux; (*d*) NaOH, THF/H_2_O, RT; (*e*) R^1^Cl, K_2_CO_3_, acetone, reflux or R^2^Cl, pyridine, 0 °C or RT

The general synthetic pathway yielding the novel derivatives **6a**, **6b**, and **8a**–**8f** is outlined in Scheme 1. 3-Hydroxy acetophenone **1** was converted into 3-acetoxy acetophenone **2** by acetylation. Treatment of substituted acetophenone **2** with bromine in the presence of AlCl_3_ in Et_2_O (Bunders et al. 2010) afforded á-bromomethylketone **3.** Subsequently, **3** and the substituted 2-aminopyridines **4**, **5** were allowed to react in the presence of sodium bicarbonate in refluxing ethanol (Fookes et al. 2008), resulting in the generation of compounds **6a** and **6b.** The deacetylation of compounds **6a** and **6b** with sodium hydroxide afforded compounds **7a** and **7b,** respectively. The akylation or acylation of **7a** and **7b** furnished the target compounds **8a**–**8f**.

**Scheme 2:**
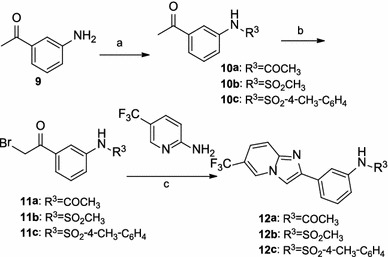
Reagents and conditions: (*a*) acetic anhydride, DMAP, anhydrous CH_2_Cl_2_, RT or R^3^Cl, pyridine, 0 °C or RT; (*b*) pyridium bromide perbromide, AcOH, RT; (*c*) NaHCO_3_, EtOH, reflux

The derivatives **12a**–**12c** were readily prepared in three steps, as illustrated in Scheme 2. 3-Aminoacetophenone **9** was first acylated with acyl chloride or sulfonyl chloride, as appropriate, to produce compounds **10a**–**10c**. Subsequent bromination of **10a**–**10c** with PBB (pyridinium bromide perbromide) in acetic acid (Yu et al. 2008) afforded compounds **11a**–**11c**, which were then cyclized with 5-trifluoro-2-aminopyridine in refluxing ethanol to give the desired derivatives **12a**–**12c**.

**Scheme 3:**
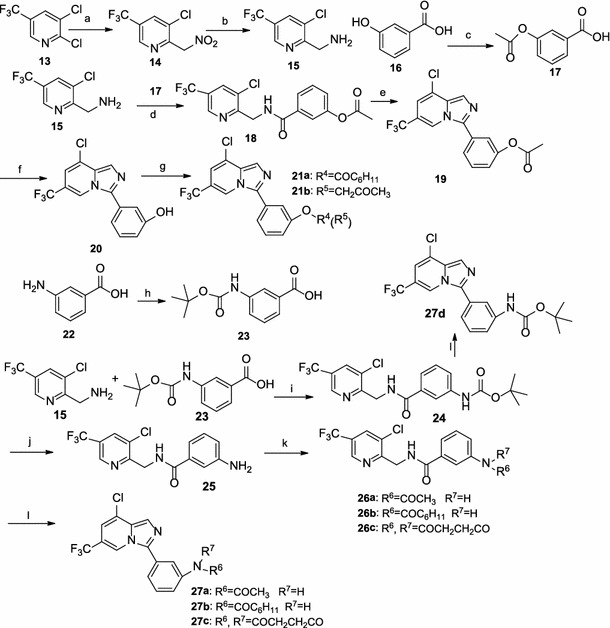
Reagents and conditions: (*a*) CH_3_NO_2_, KOH, DMSO, RT; (*b*) SnCl_2_, conc. HCl, EtOH, reflux; (*c*) acetic anhydride, reflux; (*d*) DCC, DMAP, anhydrous CH_2_Cl_2_, RT; (*e*) POCl_3_, benzene, reflux; (*f*) NaOH, THF/H_2_O, RT; (*g*) R^4^Cl, TEA, DMAP, anhydrous CH_2_Cl_2_, RT, or R^5^Cl, K_2_CO_3_, acetone, reflux. (*h*) *di*-*tert*-butyl dicarbonate, TEA, dioxane/H_2_O; (*i*) DCC, DMAP, anhydrous CH_2_Cl_2_, RT; (*j*) CF_3_COOH, anhydrous CH_2_Cl_2_, RT; (*k*) acetic anhydride, DMAP, anhydrous CH_2_Cl_2_, RT or R^6^Cl, TEA, DMAP, anhydrous CH_2_Cl_2_, RT or succimyl chloride, K_2_CO_3_, CH_3_CN, reflux; (*l*) POCl_3_, pyridine, dichloroethane, reflux

Next we synthesized the imidazo[1,5-α]pyridine derivatives **19, 21a**–**21b**, and **27a**–**27d,** as detailed in Scheme 3. The intermediate 2-aminomethylpyridine **15**, prepared from 2,3-dichloro-5-triflouropyridine **14** in two steps using a method reported elsewhere (Stolting 2004), was treated with 3-acetoxybenzoinc acid **17**, followed by cyclization in the presence of POCl_3_ in refluxing benzene (Bower and Ramage 1955) to give intermediate **19**. Then, deacylation of **19** and subsequent acylation or alkylation of the resulting compound **20** with appropriate acyl chloride or 1-chloroacetone resulted in the generation of the desired compounds **21a**–**21b**. For the synthesis of derivatives **27a**–**27d**, the first step is the amidation of intermediate **15** with benzoic acid **23** in the presence of DCC and DMAP in dichloromethane, resulting in the generation of compound **24**. Then, deprotection of **24** with trifluoroacetic acid (Mu 2001) followed by acylation of the resulting compound **25** afforded the compounds **26a**–**26c**. Finally, the amides **24** and **26a**–**26c** were cyclized in the presence of POCl_3_ and pyridine in refluxing dichloroethane (Cookson et al. 1986), resulting in the generation of the target derivatives **27a**–**27d**, respectively.

**Scheme 4:**
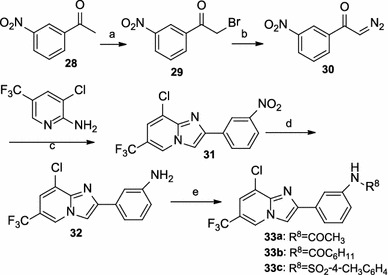
Reagents and conditions: (*a*) pyridium bromide perbromide, AcOH, reflux; (*b*) TsNHNHTs, DBU, THF, RT; (*c*) 10 mol %Cu(OTf)_2_, dichloroethane, 80 °C; (*d*) SnCl_2_, conc. HCl, EtOH, reflux; (*e*) acetic anhydride, DMAP, anhydrous CH_2_Cl_2_, RT or R^8^Cl, TEA, DMAP, anhydrous CH_2_Cl_2_, RT

Scheme 4 describes the synthesis of derivatives **33a**–**33c**. The formation of á-diazoketone intermediate **30** was achieved from á-bromomethylketone **28** by treatment with *N*,*N*′-ditosylhydrazine and DBU (Toma et al. 2007). Subsequent coupling of á-diazoketone with 3-chloro-5-trifluoro-2-aminopyridine in the presence of 10 mol % Cu(OTf)_2_ in dichloroethane (DCE) (Yadav et al. 2007) afforded substituted 2-arylimidazo[1,2-α]pyridine **31**. Reduction of **31** with stannous chloride in a refluxing mixture of ethanol and concentrated hydrochloride (Denora et al. 2008) resulted in the generation of compound **32**. Finally, the acylation of **32** with the corresponding acyl chloride afforded derivatives **33a**–**33c**.

**Scheme 5:**
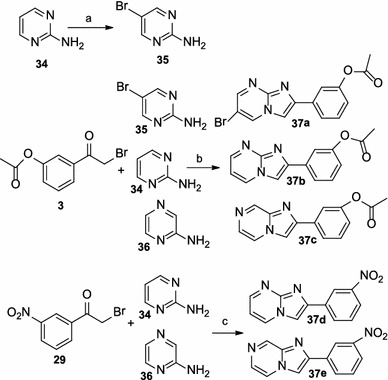
Reagents and conditions: (*a*) NBS, CH_3_CN, reflux; (*b*) dioxane, reflux or NaHCO_3_, dioxane, reflux; (*c*) EtOH, reflux

The syntheses of compounds **37a**–**37e** are detailed in Scheme 5. The preparation of intermediate **35** was achieved by bromination of 2-aminopyrimidine **34** with NBS in refluxing acetonitrile. The subsequent cyclization of **35**, 2-anmonoprimidine **34**, and 2-aminopyrazine **36** with intermediate **3** yielded the target derivatives **37a–37c**, respectively. The cyclization of 2-anmonoprimidine **34** and 2-aminopyrazine **36** with intermediate **29** yielded the target derivatives **37d–37e**, respectively.

### Biology

The compounds prepared in this study were evaluated in terms of GLP-1R agonist activity using an in vitro activation efficacy assay in CHO-K1 cells (Chen et al. 2007), and the magnitude of the responses have been compared at two concentrations of compounds used. GLP-1 (7–37) was used as the positive control and DMSO (0.1 %) was used as the negative control. Induction values represent luciferase activities driven by CRE (cAMP response element). Compounds were grouped into three series according to fused-heterocyclic ring type.

**Fig. 3:**
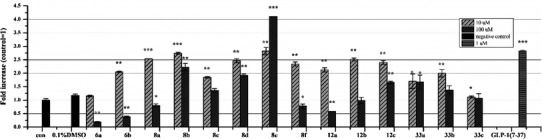
In vitro responses of compounds **6a**–**6b**, **8a**–**8f**, **12a**–**12c** and **33a**–**33c** on CHO-K1 cells at 10 and 100 μM. The cells were transfected with the pCMV6-GLP-1R and pCRE-Luc plasmids. The transfected and cultured cells were incubated with different compounds for 8 h, and luciferase activity quantified using the Steady-Glo luciferase assay system. Vertical and horizontal axes show the fold increases compared to the control and the synthesized compounds, respectively. Values shown are mean ± SD of three independent experiments. Significant difference from 0.1 % DMSO treated group: *p ≤ 0.05, **p ≤ 0.01 and ***p < 0.001

In general, the first series of compounds, **6a**–**6b**, **8a**–**8f**, **12a**–**12c**, and **33a**–**33c** (Fig. 3), based on the imidazo[1,2-α]pyridine structure and containing various substituted groups, generated higher responses than those of the second series. Compound **6b** is the model compound, in which replacement of the acetyl group with propanyl-2-one **8d**, mesyl **8e**, or tosyl **8f** resulted in a significant increase in magnitude of the response, suggesting that the hydrogen-bond donor is preferred to be this region and that the length of linker affects binding to the ago-allosteric binding site of GLP-1R. To determine whether the chlorine in imidazo[1,2-α]pyridine is essential for its activity, compounds **6a** and **8a**–**8c** were synthesized. Compounds **8a** and **8b** showed good responses similar to that of compounds **8d** and **8e** at 10 μM. However, a loss of response was observed for compounds **6a** and **8c**. The bioisosteric replacement of the ester group (**6a**, **8b**–**8c**) with an amide group (**12a**–**12c**) resulted in comparable activities; in particular, compound **12a** exhibited a two fold enhanced response. On the contrary, compounds **33a** and **33c** exhibited decreased responses. However, compound **33b**, which contains acyclohexanecarboxamide group, exhibited a moderate response. Further increases in concentration resulted in a significant drop in the responses of compounds **8a**, **6a**–**6b**, **8f**, and **12a**–**12b** to an about <1-fold increase, likely due to cytotoxicity at a high concentration (100 μM). Surprisingly, compound **8e**, which generated the highest response at 10 μM, also exhibited the greatest response at a high concentration (100 μM).

**Fig. 4:**
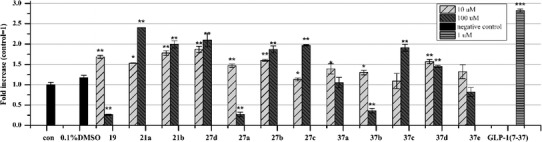
In vitro responses of compounds **19**, **21a**–**21b**, **27a**–**27d,** and **37a**–**37e** on CHO-K1 cells at 10 and 100 μM. Experimental details are described in Fig. 3. Vertical and horizontal axes show the fold increases compared to the control and the synthesized compounds, respectively. Values shown are mean ± SD of three independent experiments. Significant difference from 0.1 % DMSO treated group: *p ≤ 0.05, **p ≤ 0.01, and ***p < 0.001

In the second series of compounds, **19**, **21a**–**21b**, and **27a**–**27d** (Fig. 4), the imidazo[1,2-α]pyridine structure was changed to an imidazo[1,5-α]pyridine structure. Unfortunately, all derivatives exhibited low responses at a low concentration (10 μM). Compounds **21a**–**21b** and **27b**–**27d** showed moderate responses at 100 μM. Generally, both first- and second-series compounds with substituted ester groups showed higher responses than those with a substituted amide group.

Finally, a nitrogen atom was introduced into the six-membered fused-heterocyclic ring to evaluate the effect of electron density on activity (Fig. 4). The majority of the compounds **37a**–**37e** thus generated showed loss of responses compared to the first series. We speculated that the loss of responses might be attributable to a decreased interaction between the π-electron and the receptor.

Over the half of the synthesized compounds, the effects did not appear concentration-dependent. However the effects of the compounds on coupling the GLP-1R to the signaling way may well be concentration-dependent, but the responses measured did not appear concentration-dependent due to cytotoxicity.

**Fig. 5:**
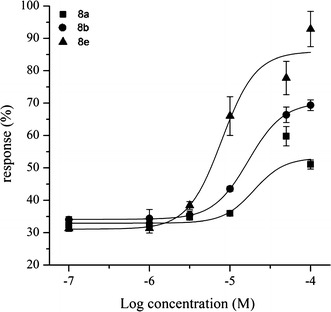
Concentration-response curves of agonists **8a**, **8b** and **8e** of GLP-1R. Experimental procedures were performed as in Fig. 3. Vertical axes show the response percentage of GLP-1 response. Values shown are mean ± SD of three independent experiments. For determined EC_50_ values see Table 1

In addition, selected compounds **8a**, **8b** and **8e** which showed >2.5-fold increases at 10 μM were assayed further to determine concentration–response curves (Fig. 5) and calculate EC_50_ values (Table 1). Compound **8e**, bearing chlorine substitution imidazo[1,2-α]pyridine ring and mesyl group of benzene ring, was found to be a potent GLP-1R agonist exhibiting an EC_50_ of 7.89 μM. Compounds **8a** and **8b**, without chlorine substitution of imidazo[1,2-α]pyridine ring, were about threefold less potent, with EC_50_ values of 20 μM and 17 μM, respectively (Table 1). Concentration–response curves of selected compounds are shown in Fig. 5. The concentration are in a range from 1 μM to 100 μM. Compounds **8b** and **8e** showed above 50 % response (**8b**, 52 %; **8e**, 58 %) at their EC_50_ values, while compound **8a** showed lower response 43 % at EC_50_ value (Fig. 5). Thus, compound **8e** may serve as a GLP-1R agonist with potential for application.

**Table 1:** Potency of agonists **8a**, **8b** and **8e** at GLP-1R

Compounds	X	Y	EC_50_ (μM)^a^
**8a**	H		19.75 ± 0.64
**8b**	H		16.96 ± 0.16
**8e**	Cl		7.89 ± 2.26

^a^Values are reported as mean ± SD

In conclusion, these new compounds, synthetic methodology developed and preliminary biological evaluation results could be helpful in further design and discovery of more potent GLP-1R agonists for the treatment of DM2.

## Electronic supplementary material

Below is the link to the electronic supplementary material.

Supplementary material 1 (DOCX 314 kb)
