# Supplementary material for: Synthesis and biological evaluation of glucagon-like peptide-1 receptor agonists
Source: Arch Pharm Res. 2013 Nov 1;37(5):588–99. doi: 10.1007/s12272-013-0253-9 (PMC4016675; doi:10.1007/s12272-013-0253-9)
Supplement: Supplementary file 1 — Supplementary material 1 (DOCX 314 kb) [file 12272_2013_253_MOESM1_ESM.docx]

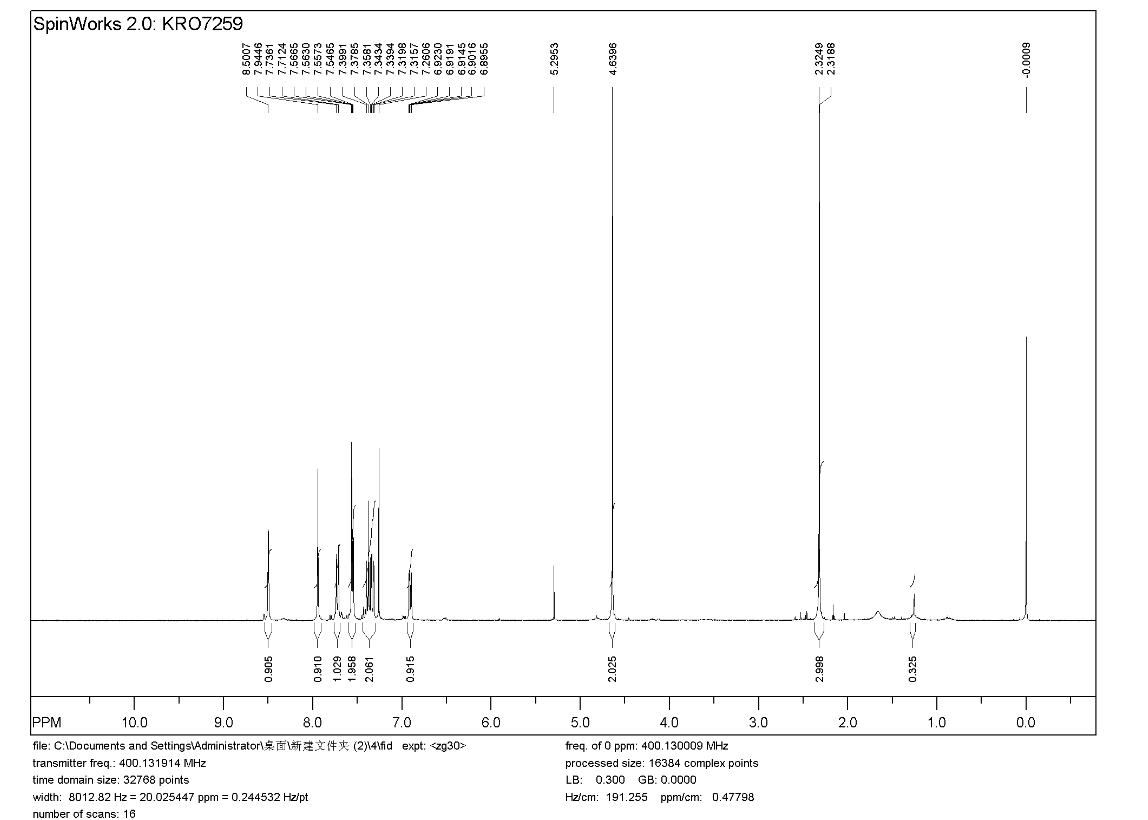


^1^H spectrum of compound **8a**


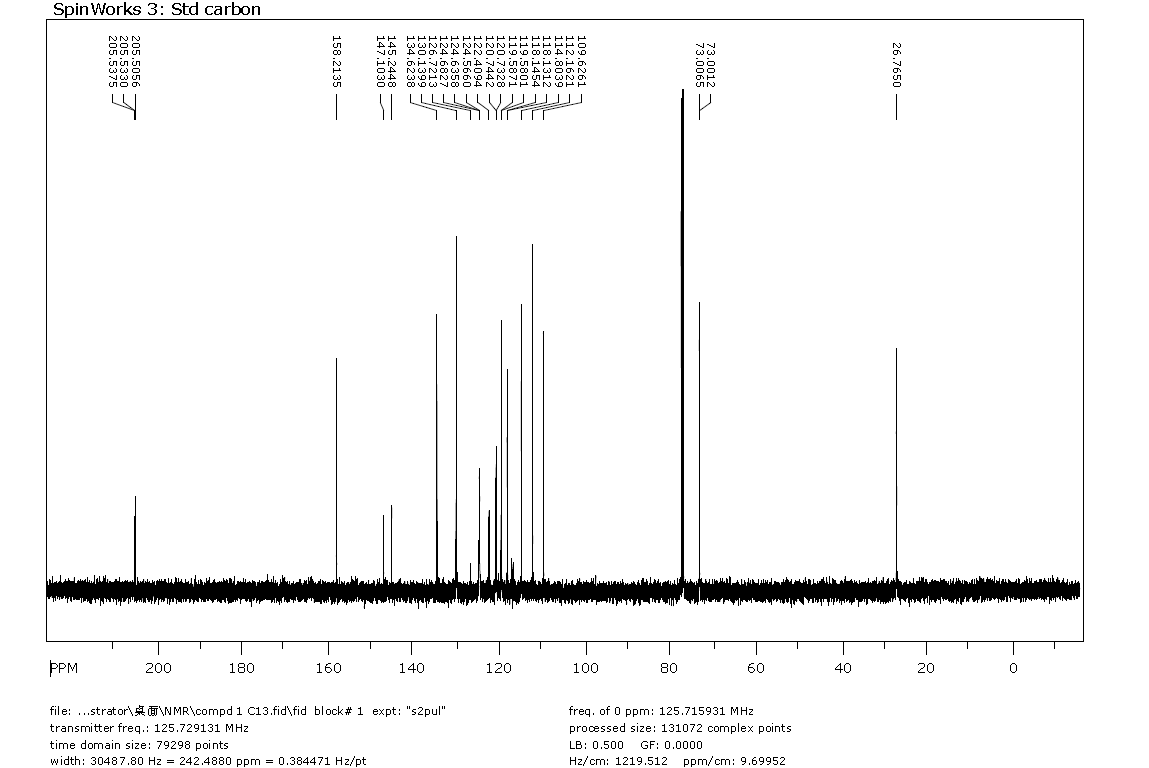


^13^C spectrum of compound **8a**

EI-HRMS spectrum of compound **8a**


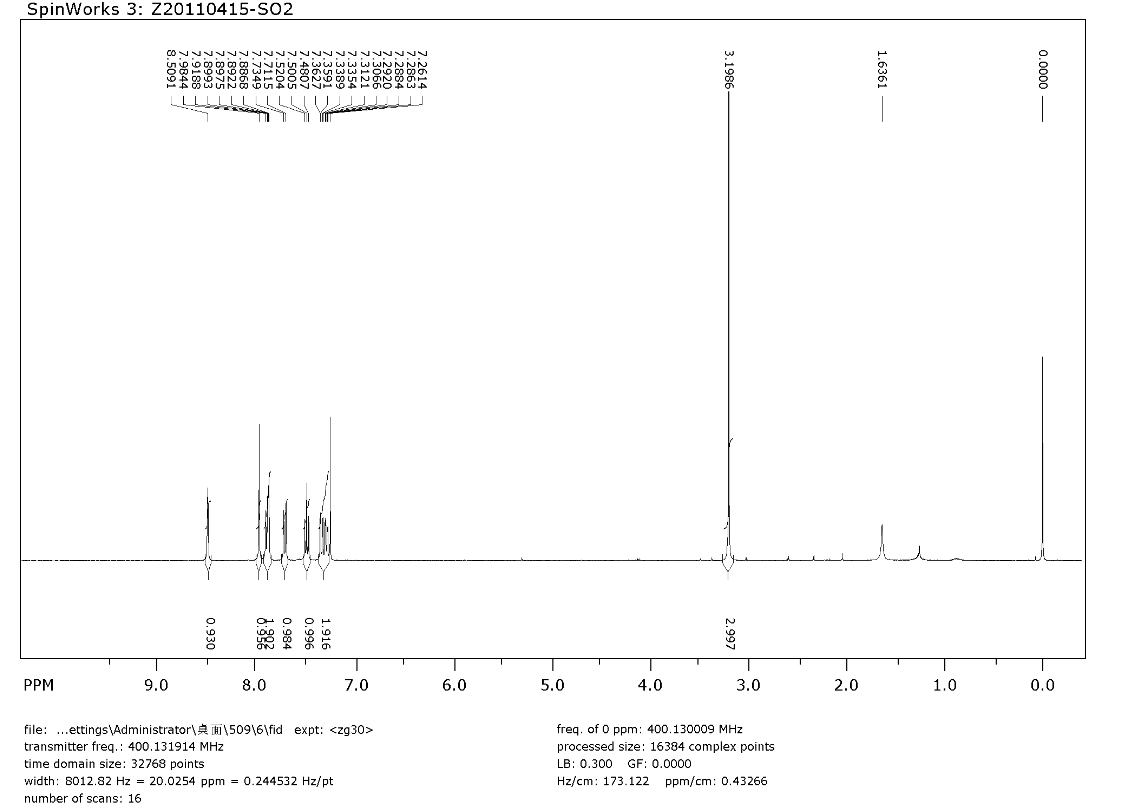


^1^H spectrum of compound **8b**


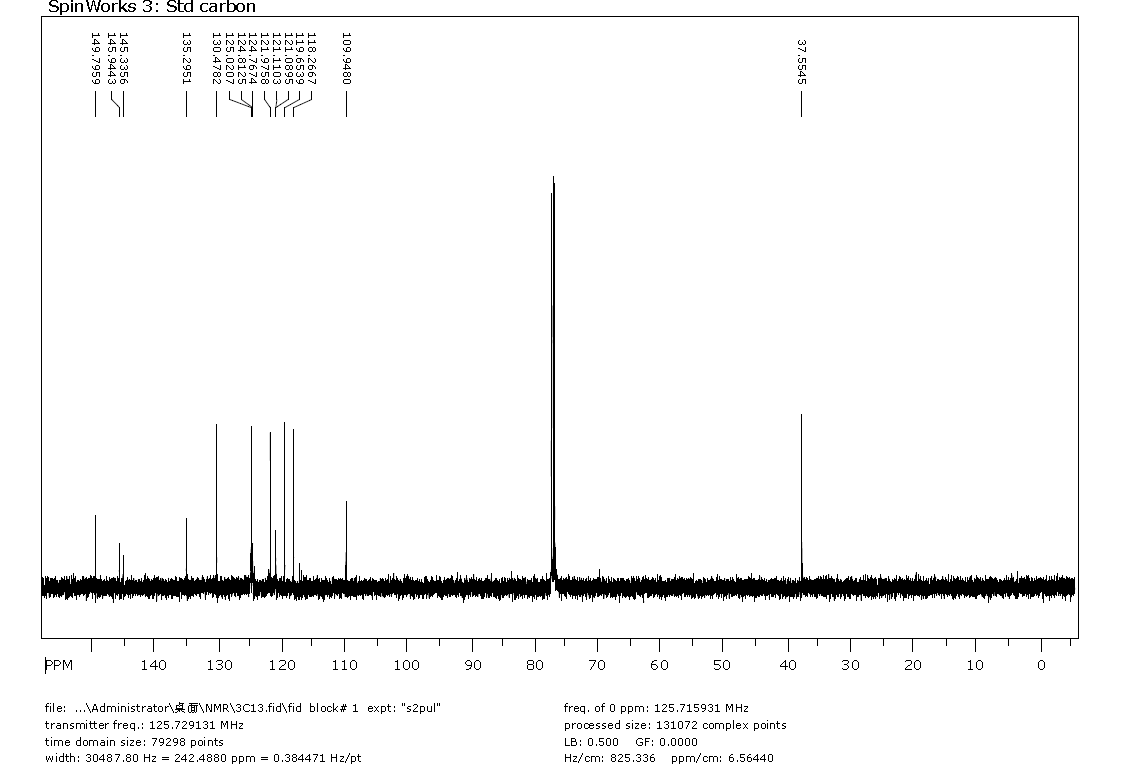


^13^C spectrum of compound **8b**

EI-HRMS spectrum of compound **8b**


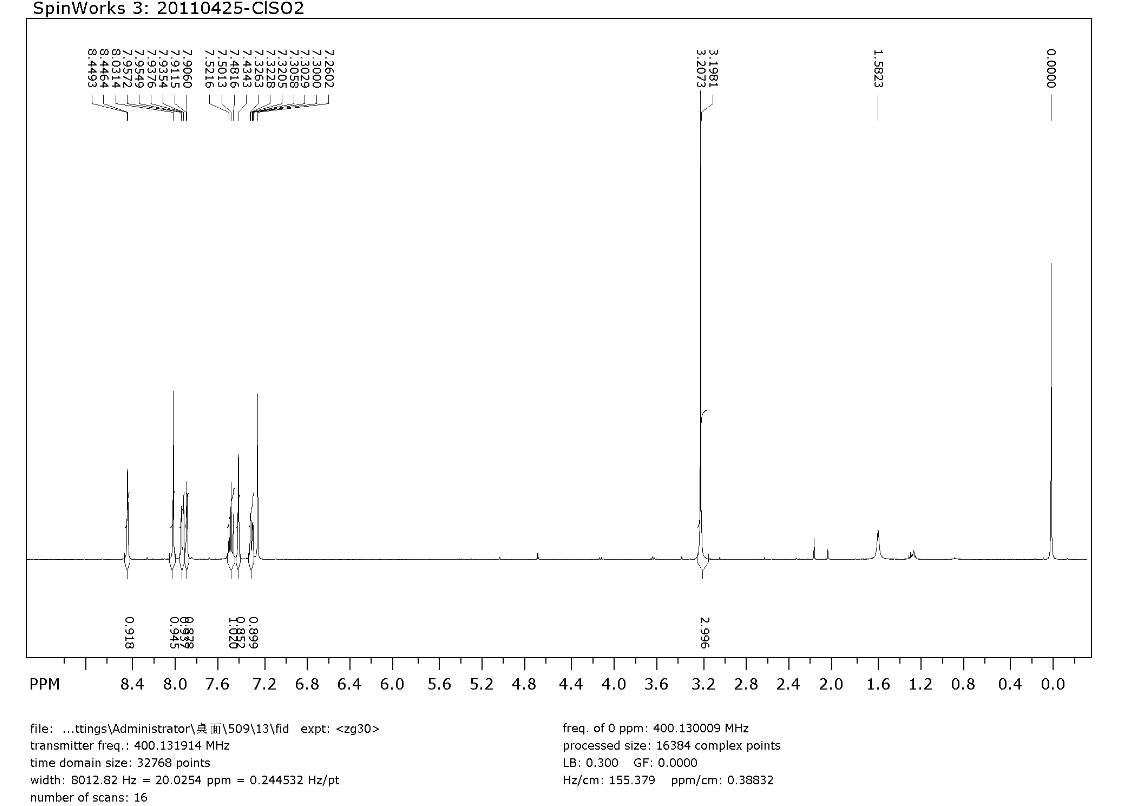


^1^H spectrum of compound **8e**


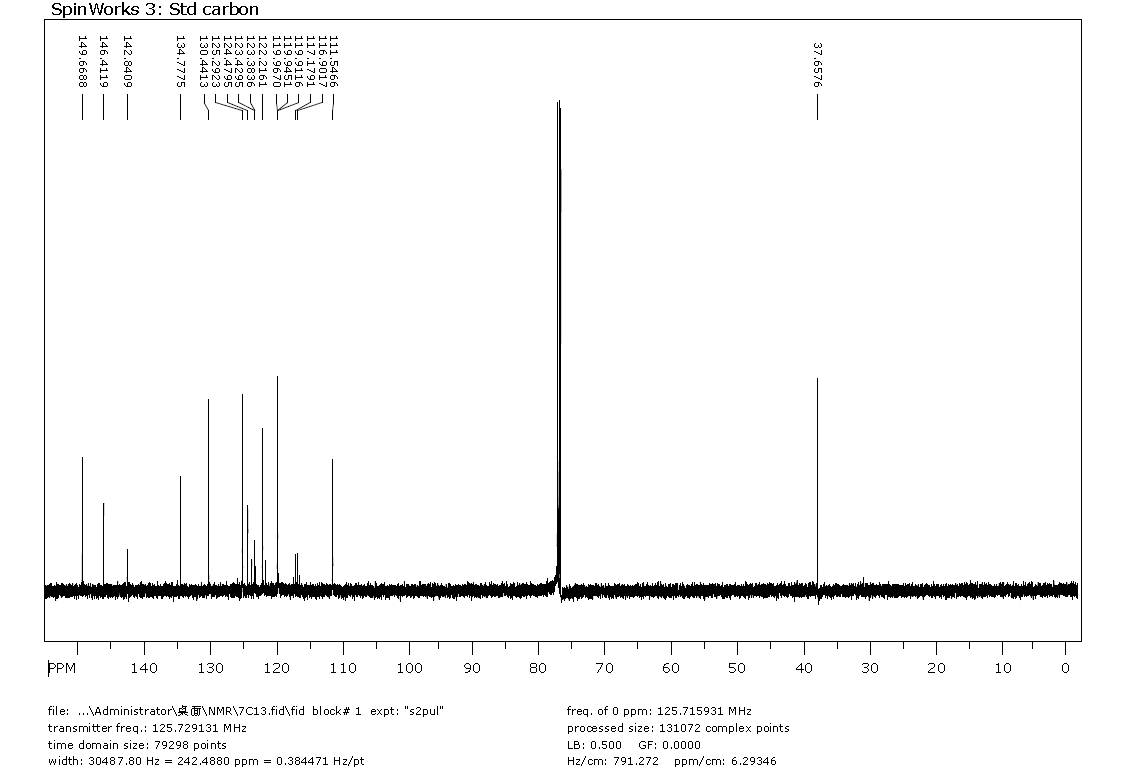


^13^C spectrum of compound **8e**

EI-HRMS spectrum of compound **8e**

**Synthesis for Intermediates**

**3-Acetylphenyl acetate (2)**

Acetic anhydride (1.22 g, 12 mmol) was added to a stirred solution of 3-hydroxyacetopnenone **1** (1.36 g, 10 mmol) and DMAP (0.12 g, 1 mmol) in anhydrous CH_2_Cl_2_ (30 mL) at room temperature. The mixture was stirred at the same temperature for 2 h. The reaction mixture was quenched with water in an ice water bath and then extracted with CH_2_Cl_2_ (3×30 mL). The combined organic phases were washed with water, 1 N HCl, and brine, dried over anhydrous magnesium sulfate, and filtered and concentrated *in vacuo* to obtain pure **2** as a white solid (1.78 g, 99%). This residue could be used without further purification. ^1^H NMR (500 MHz, CDCl_3_) δ 2.31 (s, 3H), 2.58 (s, 3H), 7.29 (d, *J* = 7.5 Hz, 1H), 7.47 (t, *J* = 7.5 Hz, 1H), 7.66 (s, 1H), 7.81 (d, *J* = 7.5 Hz, 1H). ^13^C NMR (125 MHz, CDCl_3_) δ 21.1, 26.7, 121.5, 125.8, 126.5, 129.7, 138.5, 150.9, 169.3, 197.0.

**3-(2-Bromoacetyl)phenyl acetate (3)**

Bromine (0.05 mL, 1.1 mmol) was added to a mixture of 3-acetoxyacetophenone **2** (178 mg, 1.0 mmol) and AlCl_3_ (5 mg, 0.04 mmol) in Et_2_O (10 mL) at room temperature and the reaction mixture was stirred for 1 h. The reaction mixture was washed with saturated sodium sulfite, water, and brine, dried over anhydrous magnesium sulfate, and filtered and concentrated *in vacuo* to yield crude **3** as a yellow oil (230 mg, 99%). The crude product could be used without further purification. ^1^H NMR (500 MHz, CDCl_3_) δ 2.35 (s, 3H), 4.45 (s, 2H), 7.36 (ddd. *J* = 8.0, 2.3, 1.0 Hz, 1H), 7.53 (t, *J* = 8.0 Hz, 1H), 7.72 (t, J = 2.0 Hz, 1H), 7.87 (dt, *J* = 8.0, 1.1 Hz, 1H). ^13^C NMR (125 MHz, CDCl_3_) δ 21.1, 30.7, 122.1, 126.4, 127.4, 130.0, 135.3, 151.0, 169.2, 190.3.

**3-(6-(Trifluoromethyl)imidazo[1,2-*a*]pyridin-2-yl)phenol (7a)**

To a solution of **6a** (0.90 g, 2.8 mmol) in THF (30 mL) was added NaOH (0.22 g, 5.5 mmol) in water (30 mL) with stirring for 3 h at room temperature; the reaction was monitored by TLC (hexane/ethyl acetate: 2/1) until complete. After removing THF, the resulting mixture was extracted with ethyl acetate. The combined organic phases were washed with water and brine, dried over anhydrous magnesium sulfate, and filtered and concentrated *in vacuo*. The residue was purified by silica gel column chromatography (hexane/ethyl acetate = 2/1, Rf = 0.26) to afford **7a** as a pale yellow solid (0.45 g, 58%). ^1^H NMR (400 MHz, CDCl_3_): δ 6.67 (d, *J* = 7.36 Hz, 1H), 7.15 (t, *J* = 7.6 Hz, 1H), 7.21–7.28 (m, *2*H), 7.59–7.67 (m, 3H), 8.36 (s, 1H); ^13^C NMR (100 MHz, CDCl_3_): δ 109.8, 113.3, 116.2, 117.1, 117.5, 117.6, 117.7, 121.3, 122.0, 124.5, 124.7, 129.9, 133.5, 145.1, 146.8, 157.3.

***N*-(3-acetylphenyl)acetamide (10a)**

Acetic anhydride (2.41 g, 24 mmol) was added to a stirred solution of 3-aminoacetopnenone **9** (2.73 g, 20 mmol) and DMAP (0.49 g, 4 mmol) in anhydrous CH_2_Cl_2_ (100 mL) at room temperature. The mixture was stirred at the same temperature for 2 h until the starting material had disappeared. The reaction mixture was quenched with water in an ice water bath and then extracted with CH_2_Cl_2_ (3×50 mL). The combined organic phases were washed with water, 1 N HCl, and brine, dried over anhydrous magnesium sulfate, and filtered and concentrated *in vacuo* to obtain pure **10a** as a white solid (3.52 g, 99%). This residue could be used without further purification. ^1^H NMR (500 MHz, CDCl_3_) δ 2.22 (s, 3H), 2.59 (s, 3H), 7.41 (t, *J* = 7.4Hz, 1H), 7.66 (d, *J* = 7.3 Hz, 1H), 7.99 (d, *J* = 8.1 Hz, 1H) 8.05 (s, 1H). ^13^C NMR (125 MHz, CDCl_3_) δ 24.5 26.8, 119.2, 124.2, 124.7, 129.3, 137.5, 138.8, 169.2, 198.5.

***N*-(3-acetylphenyl)methanesulfonamide (10b)**

To a solution of **9** (1.35 g, 10 mmol) in pyridine (30 mL) was added methanesulfonyl chloride (1.50 g, 13 mmol) dropwise with stirring for 2 h in an ice bath. The reaction mixture was quenched with water in an ice bath and then extracted with ethyl acetate (3×50 mL). The combined organic phases were washed with water, 1 N HCl, and brine, dried over anhydrous magnesium sulfate, and filtered and concentrated *in vacuo* to afford **10b** as a pale yellow solid (2.04 g, 96%). This residue could be used without further purification. ^1^H NMR (100 MHz, CDCl_3_) δ 2.63 (s, 3H), 3.07 (s, 3H), 7.47 (t, *J* = 7.8 Hz, 1H), 7.57 (d, *J* = 7.8 Hz, 1H), 7.69 (brs, NH), 7.76 (d, *J* = 7.4 Hz, 1H), 7.87 (s, 1H). ^13^C NMR (100 MHz, CDCl_3_) δ 26.8, 39.6, 120.1, 125.2, 130.0, 137.7, 138.3, 198.1

***N*-(3-acetylphenyl)-4-methylbenzenesulfonamide (10c)**

To a solution of **9** (1.35 g, 10 mmol) in pyridine (30 mL) was added toluenesulfonyl chloride (2.86 g, 15 mmol) dropwise in an ice bath. After stirring for 2 h at room temperature, the reaction mixture was quenched with water in an ice bath and then extracted with ethyl acetate (3×50 mL). The combined organic phases were washed with water, 1 N HCl, and brine, dried over anhydrous magnesium sulfate, and filtered and concentrated *in vacuo* to afford **10c** as a pale yellow solid (2.68 g, 93%). This residue could be used without further purification. ^1^H NMR (400 MHz, CDCl_3_) δ 2.33 (s, 3H), 2.52 (s, 3H), 7.18–7.20 (m, 2H), 7.31 (t, *J* = 7.7 Hz, 1H), 7.38 (d, *J* = 7.8 Hz, 1H), 7.63–7.70 (m, 4 H), 7.74 (brs, NH). ^13^C NMR (100 MHz, CDCl_3_) δ 21.5, 26.7, 120.6, 124.8, 125.5, 127.3, 129.6, 129.8, 135.8, 137.4, 138.0, 144.2, 197.8.

***N*-(3-(2-bromoacetyl)phenyl)-4-methylbenzenesulfonamide (11c)**

Pyridium bromide perbromide (704 mg, 2.20 mmol) was added to a solution of **10c** (585 mg, 2.02 mmol) in AcOH (100 mL) with stirring for 3 h at room temperature. The reaction mixture was poured into ice-cold water and then extracted with ethyl acetate (3×50 mL). The combined organic phases were washed with saturated aqueous NaHCO_3_, water, and brine, dried over anhydrous magnesium sulfate, and filtered and concentrated *in vacuo*. The residue was purified by silica gel column chromatography (hexane/ethyl acetate = 6/1, Rf = 0.22 ) to afford **11c** as a white crystal (578 mg, 78%). ^1^H NMR (400 MHz, CDCl_3_) δ 2.33 (s, 3H), 4.41 (s, 2H), 7.20 (d, *J* = 7.6 Hz, 2H), 7.35 (t, *J* = 7.7 Hz, 1H), 7.44 (d, *J* = 7.7 Hz, 1H), 7.67–7.74 (m, 4 H), 8.03 (s, 1H). ^13^C NMR (125 MHz, CDCl_3_) δ 21.6, 31.2, 121.0, 125.3, 126.2, 127.3, 129.9, 130.0, 134.8, 135.5, 137.7, 144.4, 191.0.

**3-Chloro-2-(nitromethyl)-5-(trifluoromethyl)pyridine (14)**

To a mixture of KOH (0.94 g, 17 mmol) in dry DMSO (30 mL) was added nitromethane (0.53 mL, 10 mmol) very slowly under a dry nitrogen atmosphere at 20°C. Stirring of the mixture at 20°C was continued for an additional 1 h. Then 2, 3-dichloro-5-trifluoromethylpyridine **13** (1.08 g, 5 mmol) was added to the reaction mixture dropwise with stirring for 3 h at room temperature. The reaction mixture was poured into ice-cold water and then extracted with ethyl acetate (3×60 mL). The combined organic phases were washed with water and brine, dried over anhydrous magnesium sulfate, and filtered and concentrated *in vacuo*. The residue was purified by silica gel column chromatography (hexane/ethyl acetate = 10/1, Rf = 0.21) to afford **14** as a pale yellow liquid (0.61 g, 51%). ^1^H NMR (400 MHz, CDCl_3_) δ 5.87 (s, 2H), 8.07 (s, 3H), 8.83 (s, 1H). ^13^C NMR (125 MHz, CDCl_3_) δ 77.0, 121.1, 123.3, 133.2, 135.0, 144.8, 150.5.

**(3-Chloro-5-(trifluoromethyl)pyridin-2-yl)methanamine (15)**

SnCl_2_.2H_2_O (18.0 g, 80 mmol) was added to a solution of nitromethylpyridine **14** (0.9 g, 4 mmol) in EtOH (50 mL) and conc. HCl (25 mL) at room temperature. The mixture was heated to reflux for 6 h and monitored by TLC (dichloromethane/methanol: 30/1) until complete. After removing EtOH, saturated Na_2_CO_3_ was added slowly until the pH value reached 10. Then the mixture was extracted with ethyl acetate (3×60 mL). The combined organic phases were washed with water and brine, dried over anhydrous calcium oxide, and filtered and concentrated *in vacuo* to afford crude **15** (0.60 g, 76%). The crude product could be used without further purification. ^1^H NMR (400 MHz, CDCl_3_) δ 4.09 (s, 2H), 7.82 (s, 3H), 8.68 (s, 1H).

**3-Acetoxybenzoic acid (17)**

A suspension of 3-hydroxybenzoic acid **16** (1.38 g, 10 mmol) in acetic anhydride (5 mL) was heated under reflux for 5 h. After cooling to room temperature, the reaction mixture was poured into iced-water (30 mL) and stirred overnight at room temperature. A white crystalline material was formed, filtered, washed with water and dried in vacuum to yield pure **17** as a white crystal (1.50 g, 83%). ^1^H NMR (500 MHz, CDCl_3_) δ 2.35 (s, 3H), 7.37 (d, *J* = 7.9Hz, 1H), 7.51 (t, *J* = 7.9 Hz, 1H), 7.85 (t, *J* = 2.0 Hz, 1H), 8.01 (d, *J* = 7.9 Hz, 1H). ^13^C NMR (125 MHz, CDCl_3_) δ 21.1, 123.5, 127.3, 127.7, 129.6, 130.8, 150.6, 169.4, 171.5.

**3-(((3-Chloro-5-(trifluoromethyl)pyridin-2-yl)methyl)carbamoyl)phenyl acetate (18)**

DCC (646 mg, 3.13 mmol) and DMAP (70 mg, 0.60 mmol) were added separately to a solution of **15** (600 mg, 2.85 mmol) and **17** (565 mg, 3.14 mmol) in anhydrous CH_2_Cl_2_ (50 mL) at room temperature. The reaction mixture was stirred for 6 h and monitored by TLC (hexane/ethyl acetate: 3/1) until complete. After filtration, the filtrate was washed with water and brine, dried over anhydrous magnesium sulfate, and filtered and concentrated *in vacuo*. The residue was purified by silica gel column chromatography (hexane/ethyl acetate = 6/1, Rf = 0.21) to afford **18** as a white solid (0.54 g, 51%). ^1^H NMR (400 MHz, CDCl_3_): δ 2.29 (s, 3H), 4.84 (s, 2H), 7.19–7.22 (m, 1H), 7.43 (t, *J* = 7.9 Hz, 1H), 7.56 (t, *J* = 1.9 Hz, 1H,), 7.69 (d, *J* = 4.4 Hz, 1H), 7.72 (s, NH), 7.91 (s, 1H), 8.70 (s, 1H).

**3-((*tert*-butoxycarbonyl)amino)benzoic acid (23)**

To a mixture of 3-aminobenzoic acid **22** (1.37 g, 10 mmol) in diaxane (50 mL) and water (25 mL) was added triethylamine (2 mL, 15 mmol), followed by di*-tert*-butyl dicarbonate (3.27 g, 15 mmol). The reaction mixture was stirred at room temperature for 24 h. Solvent was removed by rotary evaporation, and 3 N HCl (15 mL) was added dropwise to the residue. A precipitate was obtained, collected, washed with water, and dried to provide pure **23** as a white solid (2.31 g, 97%). ^1^H NMR (400 MHz, CDCl_3_) δ 1.50 (s, 9H), 7.41 (t, *J* = 7.8 Hz, 1H), 7.68 (d, *J* = 7.6 Hz, 1H), 7.79 (d, *J* = 7.7 Hz, 1H), 8.32 (s, 1H), 8.63 (brs, NH). ^13^C NMR (100 MHz, CDCl_3_) δ 28.4, 120.0, 123.2, 124.1, 129.6, 132.0, 140.9, 153.6, 167.5.

***tert*-Butyl (3-(((3-chloro-5-(trifluoromethyl)pyridin-2-yl)methyl)carbamoyl)phenyl) carbamate (24)**

Using the same method as for the preparation of **18**, starting with **15** (675 mg, 3.21 mmol), **23** (760 mg, 3.20 mmol), DCC (666 mg, 3.22 mmol), and DMAP (81 mg, 0.66 mmol), **24** was generated as a white solid (845 mg, 61%). ^1^H NMR (500 MHz, CDCl_3_): δ 1.54 (s, 9H), 4.91 (s, 2H), 6.67 (s, 1H), 7.41 (t, *J* = 7.9 Hz, 1H), 7.55 (d, *J* = 7.8 Hz, 1H), 7.67 (brs, NH), 7.73 (s, NH) 7.84 (s, 1H), 7.98 (s, 1H), 8.77 (s, 1H); ^13^C NMR (125 MHz, CDCl_3_): δ 30.9, 45.4, 119.8, 124.1, 124.3, 129.3, 132.0, 133.3, 136.8, 137.7, 141.6, 146.1, 155.3, 159.6, 169.7.

**3-Amino-*N*-((3-chloro-5-(trifluoromethyl)pyridin-2-yl)methyl)benzamide (25)**

To a mixture of **24** (100 mg, 0.23 mmol) in anhydrous CH_2_Cl_2_ (12 mL) was added CF_3_COOH (3 mL) with stirring overnight at room temperature. After removing the solvent, the residue was extracted with ethyl acetate and water. The combined organic phases were washed with 1 N NaOH, brine, dried over anhydrous calcium oxide, and filtered and concentrated to afford **25** as a white solid (74 mg, 97%). ^1^H NMR (400 MHz, CDCl_3_): δ 3.85 (brs, NH_2_), 4.89 (s, 2H), 6.83 (d, *J* = 6.8 Hz, 1H), 7.23–7.27 (m, 3H), 7.73 (s, NH), 7.97 (s, 1H), 8.76 (s, 1H); ^13^C NMR (100 MHz, CDCl_3_): δ 42.7, 113.9, 116.6, 118.1, 121.3, 124.0, 126.5, 126.9, 129.5, 130.7, 134.1, 135.3, 143.4, 146.9, 156.9, 167.6.

**3-Acetamido-*N*-((3-chloro-5-(trifluoromethyl)pyridin-2-yl)methyl)benzamide (26a)**

Acetic anhydride (14 mg, 0.13 mmol) was added to a mixture of **25** (37 mg, 0.11 mmol) and DMAP (2 mg, 0.02 mmol) in anhydrous CH_2_Cl_2_ (5 mL) with stirring for 1 h at room temperature. After removing the solvent, the residue was extracted with ethyl acetate and water. The combined organic phases were washed with 1 N HCl, saturated Na_2_CO_3_, water, and brine, dried over anhydrous magnesium sulfate, and filtered and concentrated *in vacuo* to afford pure **26a** as a white solid (40 mg, 96%). ^1^H NMR (500 MHz, CDCl_3_): δ 2.24 (s, 3H), 4.91 (s, 2H), 7.44 (t, *J* = 7.9 Hz, 1H), 7.61 (d, *J* = 7.6 Hz, 1H), 7.82 (brs, 2NH), 7.92 (d, *J* = 7.9 Hz, 1H), 7.99 (m, 2H), 8.78 (s, 1H); ^13^C NMR (125 MHz, CDCl_3_): δ 27.3, 45.4, 121.2, 125.0, 125.7, 129.3, 132.1, 133.3, 136.8, 137.5, 141.3, 146.2, 159.3, 169.7, 171.3.

***N*-((3-chloro-5-(trifluoromethyl)pyridin-2-yl)methyl)-3-(cyclohexanecarboxamido) benzamide (26b)**

Cyclohexanecarboxylic chloride (28 mg, 0.19 mmol) was added to a solution of **25** (53 mg, 0.16 mmol), TEA (19 mg, 0.19 mmol), and DMAP (4 mg, 0.03 mmol) in anhydrous CH_2_Cl_2_ (10 mL) slowly in an ice bath. After stirring for 3 h at room temperature, the reaction mixture was poured into ice water and then extracted with CH_2_Cl_2_ (3×20 mL). The combined organic phases were washed with 1 N HCl, water, and brine, dried over anhydrous magnesium sulfate, and filtered and concentrated *in vacuo* to afford **26b** as a pale yellow solid (70 mg, 99%). The crude product could be used without further purification. ^1^H NMR (400 MHz, CDCl_3_): δ 1.25 (m, 4H), 1.54 (m, 2H), 1.79 (m, 2H), 1.93 (m, 2H), 2.29 (m, 1H), 4.87 (s, 2H), 7.37 (t, *J* = 7.3 Hz, 1H), 7.56 (d, *J* = 7.3 Hz, 1H), 7.84 (s, 1H), 7.90 (d, *J* = 7.6 Hz, 1H), 7.95 (s, 1H), 8.06 (s, 1H), 8.16 (s, 1H), 8.74 (s, 1H); ^13^C NMR (100 MHz, CDCl_3_): δ 25.6, 29.5, 42.7, 46.3, 118.5, 121.2, 122.2, 123.1, 123.9, 126.5, 126.8, 129.2, 130.6, 134.1, 134.6, 139.0, 143.5, 156.7, 167.3, 175.1.

***N*-((3-chloro-5-(trifluoromethyl) pyridin-2-yl)methyl)-3-(2,5-dioxopyrrolidin-1-yl) benzamide (26c)**

Succinyl chloride (43 mg, 0.27 mmol) was added to a mixture of **25** (82 mg, 0.25 mmol), K_2_CO_3_ (37 mg, 0.27 mmol), and DMAP (3 mg, 0.03 mmol) in anhydrous CH_3_CN (10 mL) at room temperature. The reaction mixture was heated to reflux for 16 h. After cooling to room temperature, the reaction mixture was filtered, concentrated, and extracted with ethyl acetate. The combined organic phases were washed with 1 N HCl, 1 N NaOH, and brine, dried over anhydrous magnesium sulfate, and filtered and concentrated *in vacuo* to afford crude **26c** as a pale yellow solid (47 mg, 46%). The crude product could be used without further purification. ^1^H NMR (500 MHz, CDCl_3_): δ 2.94 (s, 4H), 4.92 (s, 2H), 7.49 (d, *J* = 7.9 Hz, 1H), 7.61 (t, *J* = 7.9 Hz, 1H), 7.79 (s, NH), 7.86 (s, 1H), 7.93 (d, *J* = 7.8 Hz, 1H), 7.99 (s, 1H), 8.77 (s, 1H); ^13^C NMR (125 MHz, CDCl_3_): δ 31.1, 45.4, 116.6, 119.3, 120.8, 128.1, 128.8, 132.2, 133.4, 135.0, 136.8, 138.1, 146.1, 159.3, 168.8, 178.5.

**2-Bromo-1-(3-nitrophenyl)ethanone (29)**

Pyridium bromide perbromide (12.8 g, 0.04 mol) was added to a solution of **28** (6.6 g, 0.04 mol) in AcOH (70 mL) at room temperature. The reaction mixture was heated to reflux for 7 h. After cooling to room temperature, the reaction mixture was poured into ice-cold water and then extracted with ethyl acetate (3×50 mL). The combined organic phases were washed with saturated aqueous NaHCO_3_, water, and brine, dried over anhydrous magnesium sulfate, and filtered and concentrated *in vacuo* to yield crude **29** as a yellow solid (8.0 g, 82%). The crude product could be used without further purification. ^1^H NMR (400 MHz, CDCl_3_) δ 4.49 (s, 2H), 7.74 (t, *J* = 7.9 Hz, 1H), 8.33 (d, *J* = 7.5 Hz, 1H), 8.48 (d, *J* = 7.9 Hz, 1H), 8.82 (s, 1H). ^13^C NMR (100 MHz, CDCl_3_) δ 29.9, 123.9, 128.1, 130.2, 134.5, 135.1, 148.5, 189.4.

**2-Diazo-1-(3-nitrophenyl)ethanone (30)**

DBU (8.9 mL, 60 mmol) was added dropwise to a mixture of **29** (2.92 g, 12 mmol) and *N*,*N’*-ditosylhydrazine (8.17 g, 24 mmol) in anhydrous THF (50 mL) at 0°C. Then the reaction mixture was stirred at room temperature for 10 min. After quenching by addition of saturated NaHCO_3_ solution, the product was extracted with ethyl acetate (3×50 mL). The combined organic phases were washed with water and brine, dried over anhydrous magnesium sulfate, and filtered and concentrated *in vacuo*. The residue was purified by silica gel column chromatography (hexane/ethyl acetate = 8/1, Rf = 0.25) to afford **30** as a white solid (1.18 g, 52%). ^1^H NMR (400 MHz, CDCl_3_) δ 6.05 (s, 1H), 7.68 (t, *J* = 7.9 Hz, 1H), 8.14 (d, *J* = 7.5 Hz, 1H), 8.40 (d, 7.9 Hz, 1H), 8.57 (s, 1H). ^13^C NMR (100 MHz, CDCl_3_) δ 55.3, 121.7, 127.0, 130.0, 132.5, 137.9, 148.3, 183.6.

**8-Chloro-2-(3-nitrophenyl)-6-(trifluoromethyl)imidazo[1,2-*a*]pyridine (31)**

A mixture of α‑diazoketone **30** (297 mg, 1.55 mmol), 2-aminopyridine **5** (255 mg, 1.30 mmol), and Cu(OTf)_2_ (54 mg, 0.15 mmol) in dichloroethane (30 mL) was stirred to reflux for 3 h. After cooling to room temperature, the reaction mixture was concentrated and extracted with ethyl acetate and water. The combined organic phases were washed with 6 N HCl and brine, dried over anhydrous magnesium sulfate, and filtered and concentrated *in vacuo*. The residue was purified by silica gel column chromatography (hexane/ethyl acetate = 12/1, Rf = 0.22) to afford **31** as a pale yellow solid (164 mg, 37%). ^1^H NMR (500 MHz, CDCl_3_): δ 7.48 (s, 1H), 7.66 (t, *J* = 8.0 Hz, 1H), 8.14 (s, 1H), 8.24 (d, *J* = 7.6 Hz, 1H), 8.40 (d, *J* = 7.7 Hz, 1H), 8.48 (s, 1H), 8.79 (s, 1H).

**3-(8-Chloro-6-(trifluoromethyl)imidazo[1,2-*a*]pyridin-2-yl)aniline (32)**

SnCl_2_.2H_2_O (1.95 g, 8.64 mmol) was added to a solution of **31** (0.15 g, 0.43 mmol) in EtOH (40 mL) and conc. HCl (16 mL) at room temperature. Then the mixture was heated to reflux for 4 h and monitored by TLC (dichloromethane/methanol: 30/1) until the reaction was complete. After removing EtOH, saturated Na_2_CO_3_ was added slowly until the pH value reached 10. Then the mixture was extracted with ethyl acetate (3×60 mL). The combined organic phases were washed with water and brine, dried over anhydrous calcium oxide, and filtered and concentrated *in vacuo* to afford crude **32** (0.14 g, 99%). The crude product could be used without further purification. ^1^H NMR (400 MHz, CDCl_3_): δ 3.82 (brs, NH_2_), 6.69 (d, *J* = 7.2 Hz, 1H), 7.21 (t, *J* = 7.7 Hz, 1H), 7.26 (s, 1H), 7.40 (m, 2H), 7.92 (s, 1H), 8.40 (s, 1H); ^13^C NMR (100 MHz, CDCl_3_): δ 110.9, 113.0, 115.5, 116.4, 116.5, 116.7, 119.4, 121.6, 123.2, 124.2, 124.3, 129.7, 133.3, 142.6, 146.9, 148.3.

**5-Bromopyrimidin-2-amine (35)**

NBS (1.87 g, 10.5 mmol) was added to a solution of 2-aminopyrimidien **34** (0,95 g, 10.0 mmol) in anhydrous CH_3_CN (15 mL). The mixture was heated to reflux for 12 h, and monitored by TLC (hexane/ethyl acetate: 1/1) until the reaction was complete. After cooling to room temperature, the reaction mixture was filtered. The filtrate was concentrated, dissolved in acetone, filtered again, and concentrated and dried *in vacuo*. The residue was purified by silica gel column chromatography (ethyl acetate/hexane, 33–100%, Rf = 0.23) to afford **35** as a white crystal. ^1^H NMR (400 MHz, CDCl_3_) δ 5.72 (s, NH2), 8.31 (s, 2H). ^13^C NMR (100 MHz, CDCl_3_) δ 107.1, 158.4, 161.2.
